# Influence of Conventional and Innovative Abutment Designs and Retention Mechanisms on the Biomechanics and Microgap Pattern: A 3D Finite Element Analysis

**DOI:** 10.3390/ma19010164

**Published:** 2026-01-02

**Authors:** İlayda Tunç Botello Becerra, Bahattin Alper Gültekin, Serdar Yalçın

**Affiliations:** 1 Oral Implantology Program, Institute of Graduate Studies in Health Sciences, Istanbul University, 34116 Istanbul, Turkey; 2 Department of Oral Implantology, Faculty of Dentistry, Istanbul University, 34116 Istanbul, Turkey; alperg@istanbul.edu.tr (B.A.G.);

**Keywords:** abutment, angulated, cementless, dental implant, finite element analysis, microgap

## Abstract

This study aimed to analyze the biomechanics of three abutment systems with distinct retention mechanisms and their impact on the implant–abutment interface (IAI). The finite element analysis method was used to model maxillary three-unit restorations with conventional cement-retained abutment (CRA), multi-unit abutment (MUA), and innovative cementless link-retained abutment (LRA) systems. Dental implants were positioned at 0°/0°, 15°/15°, and 25°/25° angulation combinations. Analyses were performed under 400 N vertical and 200 N oblique loading applied at a 45° angulation. The LRA system exhibited lower stress on the implants and abutments under both loading conditions, whereas the CRA system demonstrated the highest stress. In contrast, the maximum principal stresses within the peri-implant bone were the highest in the LRA system under both loading conditions. Despite greater IAI displacement in the molar region, no specific abutment system exhibited distinct superiority under different scenarios. Overall, an increase in implant angulation led to higher stress values across all parameters. The MUA and LRA systems demonstrated reduced stress concentration and more uniform load distribution compared with the CRA system under tilted implant configurations. The findings suggest that the innovative cementless LRA system may serve as a feasible alternative to conventional CRA and MUA systems, exhibiting superior biomechanical performance, particularly compared with the CRA system.

## 1. Introduction

The use of dental implants is a fundamental approach in managing partial and complete edentulism, particularly in recent years, owing to their effectiveness, high survival and success rates, and predictable clinical outcomes [1,2]. The success of dental implant therapy is basically associated with the biomechanical loading applied to the surrounding bone, the biological and structural responses of the bone to these loads, and the homogeneous distribution of functional stresses among the implant components, prosthetic restoration, and surrounding tissues [2,3,4]. The magnitude and distribution of loads are influenced by several factors, including occlusal forces, bone quality, implant surface characteristics and design, implant–abutment interface (IAI), implant positioning and angulation, soft-tissue health and thickness, parafunctional habits, and retention type of the prosthetic restoration [2,5,6]. These factors may adversely impact the early and long-term prognosis of implants by contributing to bone resorption before or after osseointegration [2].

A large number of studies focus on the preservation of peri-implant hard and soft tissues, considering the increasing use and survival rates of dental implants. However, survival alone does not necessarily indicate the overall success in clinical practice, as dental implant rehabilitation is frequently associated with mechanical and biological complications. Previous studies primarily focused on the concept of osseointegration; however, recent investigations have increasingly addressed prosthetic complications, which are among the key factors contributing to early and late implant failures [7].

Most complications are associated with micromovement, microgap, and stress accumulation at the IAI [8]. The microgap formation at the IAI can lead to bacterial leakage and cellular inflammation, thereby increasing the risk of bone resorption and soft-tissue inflammatory response [8,9,10]. The stability of the IAI influences stress distribution, and different retention systems contribute to achieving this stability [8]. The type of retention influences stress pattern within the implant, prosthetic restoration, and surrounding bone complex, thereby impacting the overall biomechanical performance of the system [11].

Conventional cement-retained abutment (CRA) and multi-unit abutment (MUA) systems have long been used in implant-supported prosthetic restorations. Screw-retained prostheses simplify retrievability and facilitate treatment in cases with limited interocclusal space. However, despite these benefits, the production process of these prostheses is more complex and expensive. A primary aesthetic limitation of screw-retained restorations is the presence of an occlusal screw-access hole, particularly in cases with aesthetics at the forefront. Furthermore, mechanical failures, including screw loosening and fracture, frequently occur in regions with concentrated stresses in screw-retained prostheses [12,13]. In contrast, cement-retained prostheses are advantageous due to their ease of achieving passive fit, favorable aesthetics, and relatively simple production procedures. Nevertheless, the presence of residual cement increases the risk of peri-implant disease [3,14]. However, innovative systems allowing for cementless retention have been introduced in recent years to minimize the limitations of traditional approaches. Within this context, a novel cementless link-retained abutment (LRA) system, particularly when used in combination with zirconia crowns, has attracted increasing attention. Unlike conventional cement-retained prostheses, the innovative link-retained design has a direct abutment to the implant connection, thereby surpassing the requirement for an auxiliary screw. The retention between the prosthetic restoration and the abutment is instead established through an additional linking component and connection (link) screw, eliminating the use of cement [11]. 

Nevertheless, abutment design and angulation are decisive in stress distribution, biomechanical performance, and maintenance of surrounding tissues within the implant-supported prosthetic complex. The use of angulated implants offers several advantages, including the preservation of anatomical structures, an increased bone–implant contact through the use of longer implants, and improved anterior–posterior distribution [15]. However, implant angulation may influence the uniform distribution of functional loads, potentially leading to increased stress at the IAI and a change in the microgap pattern [5,16]. Higher implant and abutment angulations can lead to reduced biomechanical strength and an increased incidence of screw loosening complications [15].

This study was performed to evaluate the influence of conventional and innovative abutment designs on the biomechanics and microgap pattern under different implant and abutment angulation scenarios. Although previous studies have investigated stress distribution associated with individual designs, a comprehensive evaluation integrating distinct retention mechanisms with various angulation and loading configurations has not been performed. In this context, two-implant-supported three-unit monolithic zirconia fixed prostheses were modeled using three abutment systems: CRA, MUA, and LRA. A three-dimensional (3D) finite element analysis (FEA) was conducted to examine the biomechanics of the implant–prosthetic restoration–bone structure considering vertical and oblique loading conditions.

## 2. Materials and Methods

### 2.1. Ethical Approval

The protocol of this study was reviewed and approved by the Clinical Research Ethics Committee of the Faculty of Dentistry, Istanbul University (Approval No: 2024/73). The study was financially supported by the Scientific Research Projects Coordination Unit of Istanbul University (Project Number: TDK-2024-41430).

### 2.2. 3D Model Design

A total of nine 3D finite element models were constructed, comprising three different abutment systems (CRA, MUA, and LRA). Each model represented two implants positioned at the sites of maxillary right first premolar (#14) and first molar (#16), considering three bucco-palatal angulation combinations (0°/0°, 15°/15°, and 25°/25°). Across various models, only the implant and abutment angulations, abutment systems and retention type were altered depending on the scenario, while all other components were kept constant in each model to isolate and evaluate the implications of abutment design.

In this study, the 3D geometry was configured and reconstructed as a solid structure for analysis. 3D finite element models were generated, and subsequent stress analyses of the models were performed. All simulations were carried out on a high-performance workstation. Reverse engineering procedures, 3D computer-aided design modeling and assembly of all components were completed using Blender v4.3 software (Blender Foundation, Amsterdam, The Netherlands).

Additionally, tomographic Digital Imaging and Communications in Medicine (DICOM) datasets were segmented in 3D Slicer v5.4 software (MIT AI Lab, Cambridge, MA, USA) according to appropriate Hounsfield unit thresholds (range: 426.50–3193.04) and transformed into Standard Tessellation Language (STL) files to generate 3D models representing the edentulous area corresponding to the right maxillary first premolar (#14), second premolar (#15), and first molar (#16). A maxillary cortical bone layer with a 1-mm thickness was constructed, and the trabecular bone was subsequently generated based on its internal surface. The modeling process was completed by placing all the created models spatially aligned and positioned in the correct coordinates.

**Figure 1:**
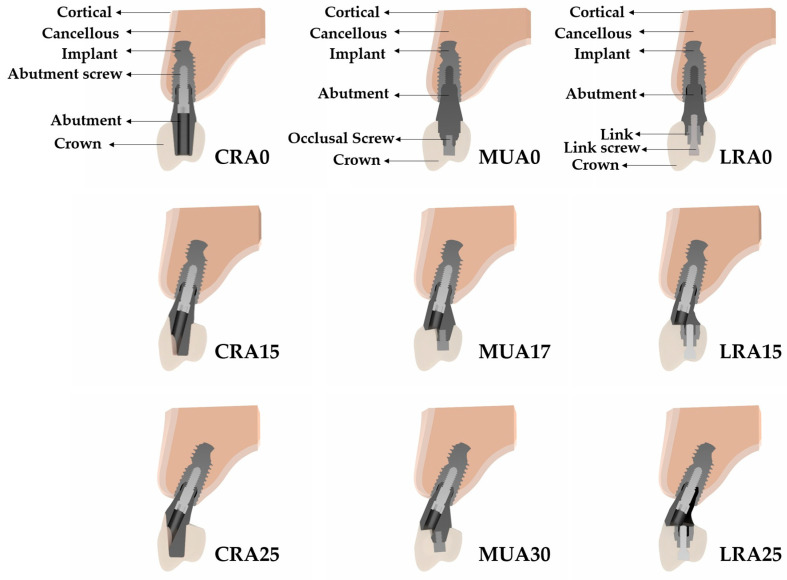
Schematic illustration of abutment systems and prosthetic components.

The implant fixture was modeled with dimensions of 4.2 mm diameter, 10 mm length, and an 11° Morse taper connection. Dental implants were positioned in the right maxillary alveolar bone models at the first premolar and first molar sites using three bucco-palatal angulation combinations: 0°/0°, 15°/15°, and 25°/25°. The implant-supported fixed prosthetic restorations incorporated three types of abutment systems: CRA with angulations of 0° (Ø4.5 mm), 15° (Ø4.5 mm), and 25° (Ø4.5 mm); MUA with angulations of 0° (Ø4.8 mm), 17° (Ø4.5 mm), and 30° (Ø4.5 mm); and cementless LRA with angulations of 0° (Ø4.5 mm), 15° (Ø4.8 mm), and 25° (Ø4.8 mm) including intermediate link components (Figure 1). Three-unit monolithic zirconia restorations were designed for all models. A uniform cement layer thickness of 50 μm was applied for the cement-retained prostheses.

Following the completion of the modeling process in Blender v4.3 software, finite element meshes for analysis and optimization of the mesh structure were established using Altair HyperMesh v2024 software (Altair, Troy, MI, USA) with highly precise triangular elements, ranging in size from 0.1 to 0.25 mm. Following surface discretization, the solid geometries were meshed using 3D tetrahedral elements. All components were connected by using common nodes to provide proper internal force transfer across contacting interfaces. Mesh density was progressively increased, and numerical convergence was assessed based on the stabilization of maximum principal stress. Further refinement beyond the selected configuration resulted in negligible changes in stress outcomes, indicating sufficient numerical convergence. The FEA was performed using the Altair OptiStruct v2024 solver (Altair).

The models used in this study are presented in Table 1 according to the dental implant and abutment angulation configurations and various abutment systems employed. The numbers of nodes and elements corresponding to each model are also provided.

### 2.3. Material Properties

Table 2 lists the numerical definitions assigned to the materials used in the present analysis. All materials were assumed to be isotropic, homogeneous, and linearly elastic.

### 2.4. Loading and Boundary Conditions

**Figure 2:**
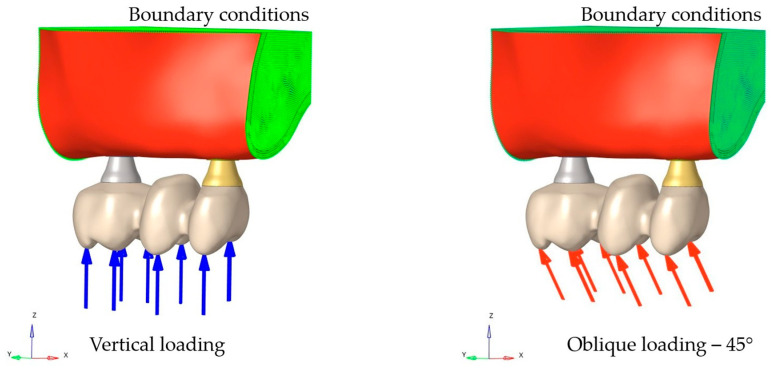
Loading and boundary conditions.

In this study, each model was subjected to a total vertical load of 400 N, distributed over four points on the lingual inclines of the cusp tips in the first molar and two points on the cusp slopes in the first premolar. Moreover, an oblique force of 200 N inclined at 45° was applied from two points on the lingual inclines of the buccal cusps in the molar and one point on the cusp slope in the premolar region (Figure 2). The applied loading conditions were determined based on a previous FEA study investigating implant-supported restorations in the maxillary molar region [16]. The forces were distributed across neighboring nodes to minimize stress singularities in the loading areas. Boundary conditions were established by constraining the distal and anterior bone nodes to eliminate displacement along all three axes.

For the analyses, fully bonded contact conditions were defined between all interacting components in the mathematical models. This method assumed that the contacting components deformed synchronously during loading, ensuring a fully bonded interaction between the surfaces.

## 3. Results

### 3.1. Maximum Principal Stress

**Figure 3:**
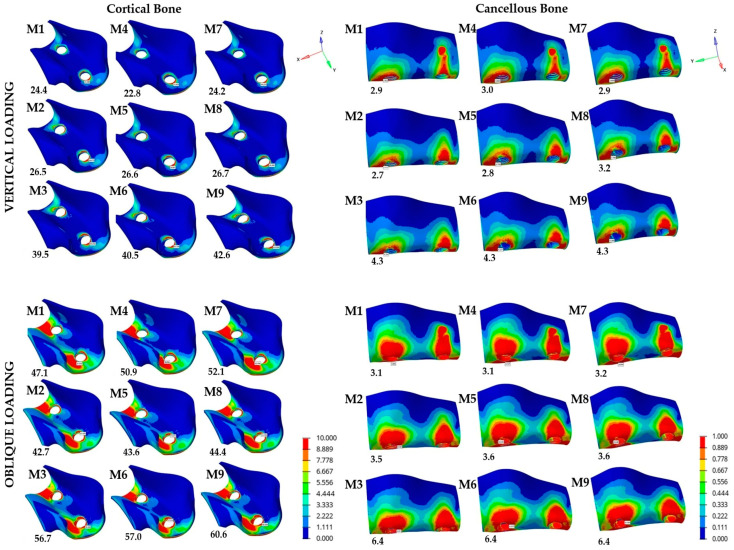
Distribution of maximum principal stresses within the peri-implant bone (MPa).

In this study, the points displaying the peak stress magnitudes in each model were identified and analyzed under vertical and oblique loading conditions. Across all models, the greatest maximum principal stresses for both cortical and cancellous bones were mainly concentrated in the first molar region (Table 3 and Figure 3).

Previous studies have reported a physiological threshold of 100–130 MPa for tensile stresses and 170–190 MPa for compressive stresses in cortical bone, whereas cancellous bone overloading has been associated with stress magnitudes exceeding 5 MPa [18]. In the present study, the cortical bone subjected to vertical loads exhibited maximum principal stress values ranging from 22.8 to 42.6 MPa. The lowest stress value was recorded in M4 (MUA0) at 22.8 MPa, whereas the highest was observed in M9 (LRA25) at 42.6 MPa (Table 3 and Figure 3). When the results were assessed based on the average maximum principal stresses for each abutment system, the cortical bone stress values were found to be 30.1 MPa (range: 24.4–39.5) in the CRA group, 30.0 MPa (range: 22.8–40.5) in the MUA group, and 31.1 MPa (range: 24.2–42.6) in the LRA group. Thus, the LRA system demonstrated the highest average maximum stress among all abutment types, whereas the MUA system exhibited the lowest. Furthermore, an increase in implant angulation led to a clear increase in stress levels within the bone tissue. The mean maximum principal stresses increased from 23.8 MPa at the 0°/0° angulation combination to 26.6 MPa at 15°/15°, and further to 40.9 MPa at 25°/25°, in the cortical bone.

Further, cancellous bone exhibited approximately 85–90% lower values of average maximum principal stress than those recorded in the cortical region. When subjected to vertical loads, the lowest stress value was recorded in M2 (CRA15) at 2.7 MPa whereas the highest value was observed in M6 (MUA25) at 4.3 MPa (Table 3 and Figure 3). The mean cancellous bone stress value for each abutment system was 3.3 MPa (range: 2.9–4.3) in the CRA group, 3.3 MPa (range: 3.0–4.3) in the MUA group, and 3.4 MPa (range: 2.9–4.3) in the LRA group. When evaluated according to angulation combinations, the mean stress value was 2.9 MPa for 0°/0°, 2.9 MPa for 15°/15°, and 4.3 MPa for 25°/25°.

When subjected to oblique loading, M2 (CRA15) revealed the lowest cortical stress at 42.7 MPa whereas the highest was recorded in M9 (LRA25) at 60.6 MPa (Table 3 and Figure 3). The mean stress value for each abutment system was 48.8 MPa (range: 42.7–56.7) in the CRA group, 50.5 MPa (range: 43.6–57.0) in the MUA group, and 52.4 MPa (range: 44.4–60.6) in the LRA group. The mean value was 50.0 MPa for 0°/0°, 43.6 MPa for 15°/15°, and 58.0 MPa for 25°/25°. Thus, the average stress levels increased with greater implant angulation, reaching the highest values for 25°/25° and the lowest values for 15°/15°, suggesting that moderately angled implant placements, which are commonly used in clinical practice, may not be disadvantageous in terms of stress pattern. Moreover, the lowest maximum principal stress value in the trabecular bone was observed in M1 (CRA0) at 3.1 MPa, whereas the highest value was recorded in M9 (LRA25) at 6.4 MPa (Table 3 and Figure 3). The mean maximum principal stress value for the CRA, MUA, and LRA systems was 4.3 MPa (range: 3.1–6.4), 4.4 MPa (range: 3.1–6.4), and 4.4 MPa (range: 3.2–6.4), respectively. However, the mean stress value for angulation combinations 0°/0°, 15°/15°, and 25°/25° was 3.1, 3.6, and 6.4 MPa, respectively.

### 3.2. Von Mises Stress on Implants and Abutments

Previous studies have reported that titanium exhibits a tensile strength ranging from 860 to 965 MPa [19].

**Figure 4:**
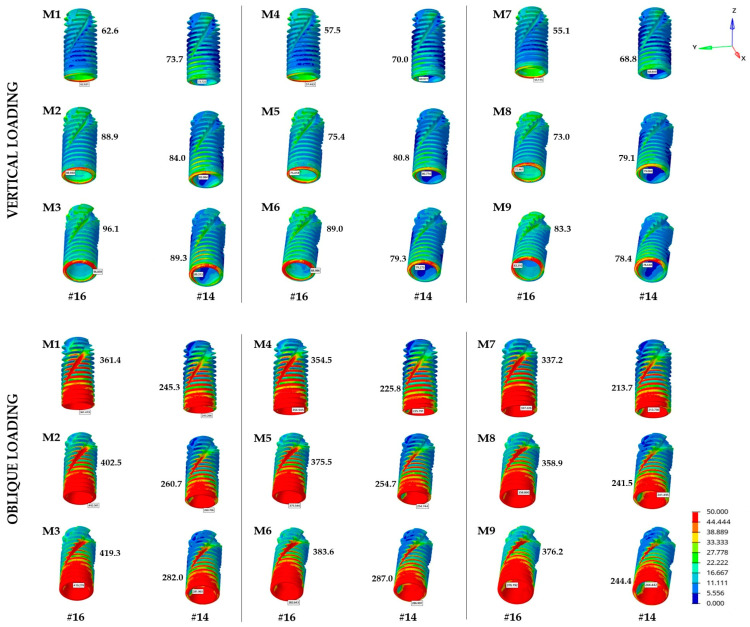
Distribution of von Mises stress on implants (MPa).

In this study, application of vertical forces resulted in von Mises stress levels between 68.8 and 89.3 MPa in the implant positioned in the maxillary first premolar region. The highest value was observed in M3 (CRA25), whereas the lowest was recorded in M7 (LRA0). For the implant positioned in the first molar region, the stress value ranged from 55.1 to 96.1 MPa, with the maximum stress again observed in M3 (CRA25) and the minimum in M7 (LRA0) (Table 4 and Figure 4). When the results were evaluated according to the abutment system, the CRA system exhibited the highest stress for implants in both regions. On average, the highest values were revealed in the CRA system (82.3 and 82.5 MPa for #14 and #16, respectively), followed by the MUA system (76.7 and 74.0 MPa) and the LRA system (75.5 and 70.5 MPa). Overall, the von Mises stresses observed in implants increased with greater implant angulation. The stress range for axially placed implants (0°/0°) varied between 55.1 and 73.7 MPa, and reached 78.4–96.1 MPa for the 25°/25° angulation combination.

The stress values observed in the implants increased significantly under oblique loads compared with vertical loads. The stress ranged between 213.7 and 287.0 MPa for the implant positioned in the maxillary first premolar region. The highest value was recorded in M6 (MUA25), whereas the lowest was observed in M7 (LRA0). The stress range was 337.2–419.3 MPa in the maxillary first molar region, with the highest stress in M3 (CRA25) and the lowest again in M7 (LRA0) (Table 4 and Figure 4). The LRA system demonstrated the lowest stress values under oblique loading, whereas the CRA system revealed the highest. Further, increasing the implant angulation resulted in a parallel increase in von Mises stress values in both implants.

**Figure 5:**
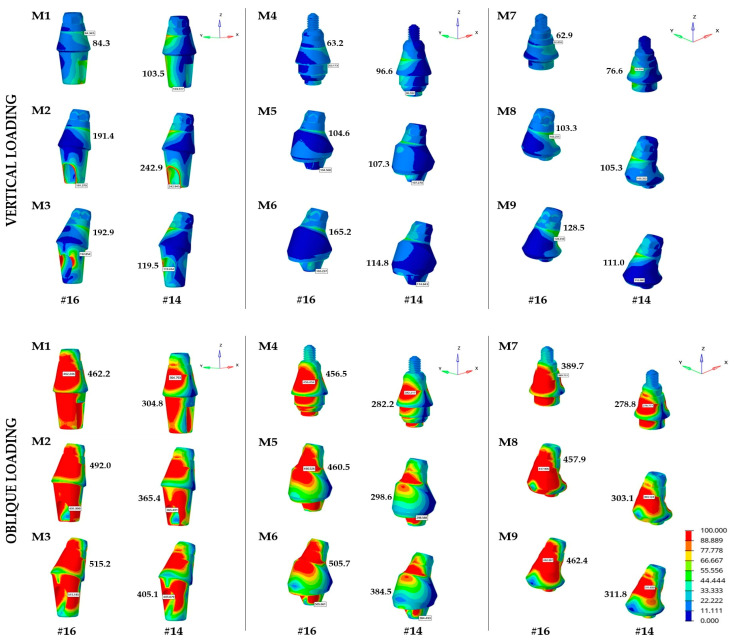
Distribution of von Mises stress on abutments (MPa).

The abutments exhibited von Mises stress magnitudes reaching a maximum of 242.9 MPa in M2 (CRA15) and a minimum of 76.6 MPa in M7 (LRA0) for the maxillary first premolar region under vertical loading. However, stress magnitudes in the first molar site ranged between the maximum and minimum values of 192.9 MPa in M3 (CRA25) and 62.9 MPa in M7 (LRA0), respectively (Table 4 and Figure 5). The mean von Mises stress value for the abutment systems in the first premolar region was 155.3 MPa for the CRA, 104.6 MPa for the MUA, and 97.6 MPa for the LRA systems. The stress values in the first molar region were 156.2, 111.0, and 98.2 MPa, respectively. Thus, the LRA system exhibited the lowest and the CRA system the highest von Mises stresses. Moreover, the stress values increased considerably with greater implant and abutment angulation. The inclined implant configurations demonstrated higher stress compared with the axially placed implants.

Overall, the findings under oblique loading revealed a prominent increase in von Mises stress values in all systems compared with vertical loading. For the implant placed in the maxillary first premolar, the highest abutment stress was observed in M3 (CRA25) at 405.1 MPa whereas the lowest was recorded in M7 (LRA 0) at 278.8 MPa. The abutment stresses in the maxillary first molar region ranged from 389.7 to 515.2 MPa, with the highest stress again seen in M3 (CRA25) and the lowest in M7 (LRA0) (Table 4 and Figure 5). The CRA system had the highest mean stress values for both implants (358.4 MPa for #14 and 489.8 MPa for #16), whereas the LRA system had the lowest (297.9 MPa for #14 and 436.7 MPa for #16). The observed increase in stress associated with angled abutments, in conjunction with higher implant angulations, implied that the use of such components might constitute less favorable stress distribution compared with straight abutments.

### 3.3. IAI Displacement

**Figure 6:**
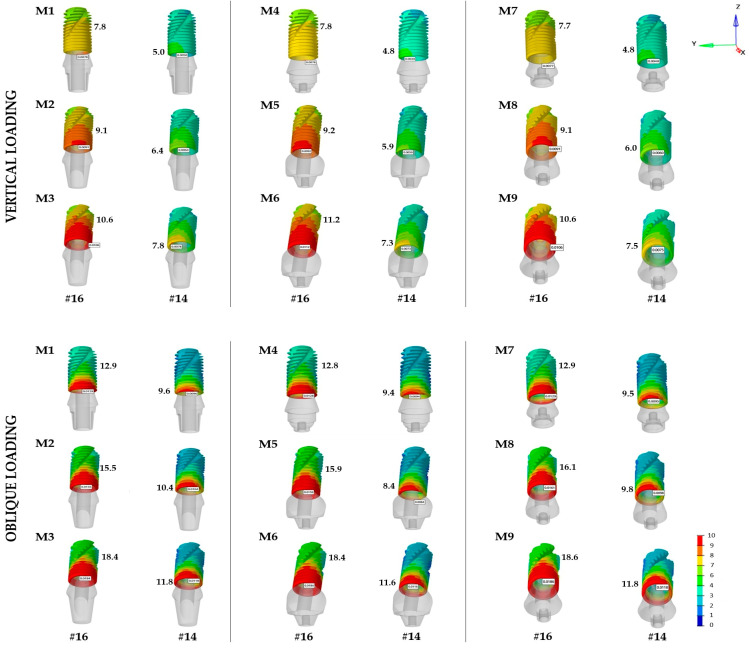
Distribution of displacement at the IAI (µm).

Intergroup comparisons indicated that the microgap pattern observed at the IAI was influenced by both the direction of forces and the implant angulation. Overall, the extent of displacement at the IAI was fundamentally greater under oblique loads than under vertical loads. Moreover, it was consistently greater in the molar region under both loading directions. The highest displacement formation under vertical loading was determined in M6 in the molar region (MUA25) as 11.2 µm, whereas the greatest value under oblique forces was observed in M9 (LRA25) as 18.6 µm. The lowest displacement as 4.8 µm was recorded in M7 and M4 under vertical loading and 8.4 µm in M5 under oblique loading at the premolar site (Table 4 and Figure 6).

Under vertical loading, the MUA (mean: 6.0 µm) and LRA (mean: 6.1 µm) systems exhibited similar and relatively lower values at the premolar site, whereas the CRA (9.2 µm) and LRA (9.1 µm) systems showed analogous averages at the molar site. Also, the MUA system presented a relatively higher mean value (9.4 µm). The highest mean microgap in the premolar region was found in the CRA system (10.6 µm), and the lowest in the MUA system (9.8 µm), under oblique loading. All systems exhibited similar mean values in the molar region: CRA (15.6 µm), MUA (15.7 µm), and LRA (15.9 µm). Overall, increased implant angulation led to a significant increase in displacement and microgap pattern along all systems.

## 4. Discussion

This study assessed the biomechanical behavior of the implant–bone–prosthetic restoration complex and microgap pattern at the IAI in relation to various abutment designs and retention mechanisms. The findings demonstrated that the conventional CRA and MUA systems, as well as the innovative cementless LRA system, significantly influenced the biomechanics and the displacement at the IAI. In addition, the present analysis enabled a direct comparison of different systems under varying implant angulation and loading conditions within a numerical framework.

The von Mises stress analysis in this study demonstrated that the LRA system displayed lower stress profile in the implant and abutment components under vertical and oblique loading conditions, whereas the conventional CRA system demonstrated higher stress concentrations compared with the other systems (Table 4 and Figure 4 and Figure 5). This outcome might be attributed to the structural rigidity of the IAI and the greater number of components within the abutment–prosthetic restoration complex. The presence of an additional link component in the LRA system is compatible with this relationship and aligns with previous findings of Lee et al. [11]. The applied loads were distributed more homogeneously along the implant and abutment components in the LRA system, resulting in reduced localized stress concentrations. These findings might be attributed to the ability of the additional link component to function as a mechanical buffer within the loading pathway. Instead of transferring occlusal forces directly to the implant neck, the additional component may modify the transmission by absorbing part of the load and dissipating it over a wider contact area, thereby minimizing the localized stress peaks. The link also reduces stress concentration in the connection region by introducing a more gradual geometric transition between the implant and the suprastructure. Abutment systems with a larger number of components often exhibit more favorable biomechanical behavior, as the stresses are distributed along the intermediate abutment parts and screws before attaining the bone–implant interface [3,11,20]. This condition, though depending on the compatibility and tolerance between the components, can also result in a limited degree of micromovement, thereby minimizing the load transmitted to the alveolar bone [11,21]. In contrast, conventional CRA systems cause localized stress concentrations due to their simpler structure and fewer components. The conventional CRA system, which uses a one-piece CRA, remains one of the most widely used clinical approaches because of its high feasibility [22].

Further, the highest stress levels were localized mainly in the neck of the implants and in the IAI of the abutments in all systems in the present study. This distribution was consistent with the observed biomechanical behaviors, as the crestal region is the first site to receive and transfer occlusal loads to the surrounding bone. The relatively narrow cross-section at the implant neck, combined with the rigidity of the cortical bone in this region, results in prominent bending moments and concentrated stress levels. Additionally, the IAI demonstrates a geometric discontinuity where modifications in diameter, material contact, and microgaps can further expand stress localization. These factors may collectively refer to peak stress values at the implant neck and IAI. Numerous studies examining the biomechanical stress pattern of this system have reported that stresses tend to intensify at the implant neck and IAI of the abutment body [22,23,24,25]. However, the value of these stresses is also influenced by loading conditions and the defined properties of the materials involved [22].

Additionally, the outcomes of this study aligned with previously reported findings. The CRA system demonstrated the greatest stress values, whereas the MUA system displayed near-optimal and comparable values to the LRA system under certain conditions. A portion of the stress was dispersed among the prosthetic restoration components in both the LRA and MUA systems, consequently minimizing the stress transmitted to the implant–bone interface. This finding suggests that these systems, especially under high-angled implant placements, may offer more clinically reliable alternatives in terms of biomechanical performance. Several previous studies comparatively focused on the success rates and complications associated with cement-retained and multi-unit prosthetic restorations [22,26]. Erdoğdu et al. [26] compared the fatigue performance of three-unit cement-retained and multi-unit restorations under different implant angulation conditions. The results indicated that MUA prostheses exhibited superior performance compared with CRA prostheses. The findings also emphasized that the selection of retention type became more critical with greater implant angulation. MUA prostheses significantly contributed to reducing stress values under high-angulation conditions.

The findings of the present study revealed that an increase in angulation generally led to higher stress and strain levels in both the implant components and peri-implant bone under vertical and oblique loading conditions. Particularly, all systems displayed the highest stress levels within the implant–bone–prosthetic restoration complex for the 25°/25° combinations (Table 3 and Table 4 and Figure 3, Figure 4 and Figure 5). This finding is attributed to the fact that greater angulation increases the bending moment at the implant neck, thus negatively impacting stress distribution. The MUA and LRA systems appeared to compensate for the effects of angulation more effectively than the CRA system. Several systematic reviews have indicated that tilted implants exhibit comparable marginal bone loss and survival rates to those of axially placed implants [16,27]. However, most of these evaluations were performed on full-arch fixed prostheses. Clinical studies investigating fixed partial restorations have reported greater peri-implant bone loss around tilted implants compared with axial implants, despite no significant differences in implant survival rates [16,28]. Overall, the findings of these clinical studies were consistent with the present FEA, yet further clinical investigations are required to clarify the impact of increased stress caused by implant angulation, particularly in abutment systems with different retention mechanisms and components. Additionally, another FEA evaluating the biomechanical effects of tilted implants in the posterior maxilla reported that angled implant placement increased stress levels compared with axial placement. Despite the elevated stress values, this approach was highlighted as a feasible alternative to additional surgical procedures in the maxillary sinus region [29].

An FEA of de Faria et al. [16] examined the biomechanical effects of splinted and non-splinted three-unit implant-supported prostheses using tilted implants with external hexagon connections in the posterior maxilla. It reported higher stress levels in the cortical bone of tilted implants compared with axially placed ones. Also, tilted implants led to concentrated stress zones, particularly in the molar region compared with the premolar region [16]. The present study also reported that an increase in implant angulation under vertical loading led to elevated stress magnitudes in the cortical bone. The highest stress levels were identified for the 25°/25° combination under oblique loading, whereas the 15°/15° configuration had lower stress values compared with the 0°/0° combination (Table 3 and Figure 3). However, despite an increase in stress levels with higher implant and abutment angulation combinations, the 15°/15° configuration produced lower maximum principal stresses than the 0°/0° configuration under certain conditions. This outcome suggested that the interaction between the implant–bone complex and the loading direction was more complex than a simple linear interaction. The occlusal force may not be perfectly aligned with the implant axis in the 0°/0° configuration, which may contribute to intensified localized stress concentrations in the crestal area. However, a moderate angulation, such as a 15°/15° configuration applied in the present study, may position the implant more favorably relative to the direction of applied load, thereby enabling a more uniform stress distribution. Given that stress (MPa) is defined as force normalized to surface area, dissipation of occlusal loads over an expanded effective contact area may account for this more uniform distribution and the associated reduction in peak local stress values. As a result, lower average stress levels in the cancellous bone under vertical loads and in the cortical bone under oblique loads were observed in the 15°/15° combination. Nevertheless, further analysis incorporating various scenarios may be required to clarify this relationship and to assess the reproducibility of these findings. Additionally, the mean maximum principal stresses observed in both the cortical and cancellous bone were relatively similar across all systems in the present study. However, when the system averages were compared in a relative manner, the highest values under both loading directions were recorded in the LRA system whereas the lowest maximum principal stress in the cortical bone under vertical loading was recorded in the MUA system. All other results indicated a slight biomechanical advantage in favor of the CRA system. These findings might be attributed to distinctions in load transmission associated with the retention mechanism of the LRA system, whereby reduced stress concentrations within the implant components are accompanied by higher stress levels in the peri-implant bone.

Furthermore, regardless of the retention type or angulation combination, all parameters demonstrated higher stress values under oblique loads compared with vertical loads. These results suggest that axial forces are transmitted predominantly along the long axis of implants, whereas oblique forces introduce a lateral component generating distinct bending moments, thereby increasing stress concentrations in the crestal region. Numerous studies evaluating biomechanical performance have applied both vertical and oblique loadings simultaneously [6,30]. A study applying vertical and 30° oblique loadings to the occlusal surfaces of various implant types, specifically at the buccal cusp tips and central fossa points, showed that the stress concentration under oblique loading was approximately 3.5 times greater [30]. In the present study, a total vertical force of 400 N and a 45° oblique force of 200 N were applied to the premolar and molar regions. As a result, the stress values observed in the implants, abutments, and peri-implant bone under oblique loading were consistently higher than those under vertical loading. Additionally, oblique forces induced a greater amount of displacement at the IAI (Table 3 and Table 4).

Previous studies have reported that the misfit between implant components can lead to microgap formation, thereby increasing the risk of mechanical and biological complications [10,31,32]. Therefore, evaluating such discrepancies using reproducible and precise methods is essential to guide clinical applications. Optical microscopy, radiographic analysis, and scanning electron microscopy are commonly used techniques for assessing microgaps. Although several studies have reported clinically acceptable microgap values, no clear consensus exists regarding the exact threshold. Generally, a clinical microgap between 10 and 150 µm is considered acceptable [10,33]. An International Team for Implantology (ITI) treatment report indicated that an implant had at least eight interfaces including the abutment-screw hole and the IAI, and that it was impossible to completely eliminate microgaps between these interfaces due to the precision limitations inherent in the manufacturing process [22,34]. Recent conical IAIs provide improved resistance to microgap formation and bacterial colonization; however, they still cannot entirely prevent the presence of these gaps [35]. A recent study of Anniwaer et al. [22] evaluating the mechanical behavior and microgap characteristics of CRA, hybrid, and MUA prostheses in anterior restorations reported that the MUA group had the most favorable microgap pattern after cyclic loading.

In the present study, the results showed that the displacement at the IAI in all systems remained below 10–12 µm under vertical loading. In contrast, oblique loading resulted in a significant increase in values, reaching up to 18.6 µm, particularly in the first molar region. Additionally, the effect of greater implant angulation led to a distinct increase in all systems (Table 4 and Figure 6). This increase might be related to the altered load transfer and higher rotational moment induced by greater implant angulation and oblique forces at the IAI. Although the IAI displacement remained within clinically acceptable ranges, microgap formation and micromovements at the IAI have been reported as potential sources of bacterial leakage, which may contribute to peri-implant inflammation and marginal bone loss [10]. Accordingly, clinical parameters influencing the stability of the IAI, including retention type, implant angulation, and loading conditions should be considered in line with these findings. Consistent with the present study, Lee et al. and Nie et al. also reported that oblique loading increased the risk of microgap formation and microleakage, whereas vertical loading promoted a more uniform stress distribution [5,13].

In this study, nine experimental models were established to evaluate three different abutment systems and three implant placement configurations using the FEA method. FEA is a valuable and widely used tool for analyzing dental restorations, biomaterials, and material designs, as well as assessing stress distribution under various loading conditions. In dental biomechanics research, this method enables quantitative and comparative evaluation of stress distribution within complex structures. Within the scope of the present study, it was assumed that all components were perfectly bonded during the modeling process, and the material properties were considered isotropic and linearly elastic. Despite providing valuable biomechanical insights guiding clinical applications, these findings should be interpreted within the limitations of the FEA method.

Additionally, in the present study, the use of a single prosthetic restoration configuration limited to the posterior maxillary region, along with constant implant geometry and connection type, necessitates cautious interpretation of the results. Factors such as the biological variability of cortical and cancellous bones, the absence of cyclic loading conditions simulating masticatory patterns, and the simplifications in geometric modeling indicate the need for further clinical and in vitro studies that systematically assess variations in implant-, restoration-, and patient-related parameters, including combined three-dimensional implant angulations, to validate and strengthen the outcomes of the present analysis.

## 5. Conclusions

Given the limitations of this study, the innovative cementless LRA system demonstrated lower stress concentrations on the implant components compared with the conventional CRA and MUA systems. However, increased implant and abutment angulation led to higher stress concentrations and greater microgap formation across all systems. Furthermore, the LRA system exhibited overall biomechanical performance comparable to that of the MUA system and more favorable than that of the CRA system, particularly under various tilted implant configurations modeled in the present study. Based on these findings, it can be concluded that innovative abutment systems may serve as viable alternatives to conventional systems under specific conditions.

## Figures and Tables

**Table 1:** Description of the models.

Model	Abutment System	Implant Angulation		Abutment Angulation		Node Count	Element Count
		#14	#16	#14	#16		
M1	CRA0	0°	0°	0°	0°	459,570	1,822,708
M2	CRA15	15°	15°	15°	15°	459,977	1,835,563
M3	CRA25	25°	25°	25°	25°	453,172	1,802,892
M4	MUA0	0°	0°	0°	0°	407,135	1,642,354
M5	MUA17	15°	15°	17°	17°	448,949	1,818,038
M6	MUA30	25°	25°	30°	30°	452,431	1,828,541
M7	LRA0	0°	0°	0°	0°	411,267	1,652,857
M8	LRA15	15°	15°	15°	15°	457,073	1,840,550
M9	LRA25	25°	25°	25°	25°	457,124	1,836,625

M: Model; #14: First premolar; #16: First molar.

**Table 2:** Material properties of the finite element model.

Component	Modulus of Elasticity (MPa)	Poisson’s Ratio (v)	Reference
Titanium	110,000	0.33	[5,17]
Zirconia	210,000	0.35	[17]
Cement	10,760	0.35	[5]
Cortical Bone	13,700	0.30	[5,17]
Cancellous Bone	1370	0.30	[17]

**Table 3:** Distribution of maximum principal stresses within the peri-implant bone.

	Vertical Loading (MPa)		Oblique Loading (MPa)	
Model	Cortical Bone	Cancellous Bone	Cortical Bone	Cancellous Bone
M1	24.4	2.9	47.1	3.1
M2	26.5	2.7	42.7	3.5
M3	39.5	4.3	56.7	6.4
M4	22.8	3.0	50.9	3.1
M5	26.6	2.8	43.6	3.6
M6	40.5	4.3	57.0	6.4
M7	24.2	2.9	52.1	3.2
M8	26.7	3.2	44.4	3.6
M9	42.6	4.3	60.6	6.4

**Table 4:** Distribution of von Mises stress and displacement on implants, abutments, and IAI.

		Vertical Loading			Oblique Loading		
Model	Implant No	Implant (MPa)	Abutment (MPa)	IAI (µm)	Implant (MPa)	Abutment (MPa)	IAI (µm)
M1	#14 #16	73.7 62.6	103.5 84.3	5.0 7.8	245.3 361.4	304.8 462.2	9.6 12.9
M2	#14 #16	84.0 88.9	242.9 191.4	6.4 9.1	260.7 402.5	365.4 492.0	10.4 15.5
M3	#14 #16	89.3 96.1	119.5 192.9	7.8 10.6	282.0 419.3	405.1 515.2	11.8 18.4
M4	#14 #16	70.0 57.5	96.6 63.2	4.8 7.8	225.8 354.5	282.2 456.5	9.4 12.8
M5	#14 #16	80.8 75.4	107.3 104.6	5.9 9.2	254.7 375.5	298.6 460.5	8.4 15.9
M6	#14 #16	79.3 89.0	114.8 165.2	7.3 11.2	287.0 383.6	384.5 505.7	11.6 18.4
M7	#14 #16	68.8 55.1	76.6 62.9	4.8 7.7	213.7 337.2	278.8 389.7	9.5 12.9
M8	#14 #16	79.1 73.0	105.3 103.3	6.0 9.1	241.5 358.9	303.1 457.9	9.8 16.1
M9	#14 #16	78.4 83.3	111.0 128.5	7.5 10.6	244.4 376.2	311.8 462.4	11.8 18.6

## Data Availability

The original contributions presented in this study are included in the article; further inquiries can be directed to the corresponding author.
